# Usefulness of the Serial Measurement of Vi Antibodies

**DOI:** 10.1093/cid/cix1151

**Published:** 2018-01-17

**Authors:** Keith P Klugman

**Affiliations:** 1 School of Pathology, University of the Witwatersrand, Johannesburg, South Africa; 2 School of Public Health, Emory University, Atlanta, Georgia; 3 Bill & Melinda Gates Foundation, Seattle, Washington

**Keywords:** typhoid fever, Vi antibodies, conjugate Vi vaccine, vaccine efficacy

**(See the Major Article by Voysey and Pollard on pages 18–24.)**

In a secondary analysis [1] of a published immunogenicity trial [2] comparing Vi capsular polysaccharide vaccine with a similar dose (25 µg) conjugated to tetanus toxoid, Voysey and Pollard have succeeded in not only establishing an argument that serial measurement of Vi antibodies are a marker of typhoid exposure but have also paved the way for future conjugate typhoid vaccines to be evaluated in endemic settings at a fraction of the recruitment required for traditional typhoid efficacy trials.

The key to their observations came from extending the analysis of the data from the trial beyond the initial immunogenicity timepoint of 6 weeks postimmunization, to consider further time points 18 months and 2 years after the initial vaccines were administered. Antibody decay was analyzed using a Gaussian mixture model during 2 time periods (6 weeks to 18 months; and 18–24 months) that assumed quite reasonably that a single Vi immunization would lead to a decay of antibody in the first period and that anyone with higher levels of antibody at 18 months than at 6 weeks postimmunization, had had an additional exposure to Vi antigen. Further, subjects with an increase in Vi antibody, or indeed a sharp decay in Vi antibody between 18 months and 24 months had likewise been exposed to Vi antigen either in the intervening months (rise in antibody) or shortly before 18 months (rapid fall in antibody between 18 and 24 months).

The authors’ posit that these Vi antigen exposures are symptomatic or asymptomatic episodes of typhoid infection. Is this reasonable? As they point out in their discussion, the Vi antigen has been found in gut commensals such as *Citrobacter*, so the proof of their contention may lie in studies that associate Vi antibodies specifically with *Salmonella* Typhi exposure in endemic areas. Chronic typhoid carriers have S. Typhi antibodies orders of magnitude greater than those reported post acute infection or postimmunization [3]; the change in antibody concentrations according to their definition, found by Voysey and Pollard to define typhoid infection in this study, across 8 trial sites in India occurred in a remarkable 21% of subjects, for whom serology was available, over 2 years. Could so many subjects have been exposed to typhoid infection in a 2-year period? If so, then the vast majority of typhoid infections are asymptomatic as typhoid clinical attack rates, measured by hospitalization, rarely exceed 1% of subjects at risk per year, even in highly endemic areas.

Few long-term follow-up studies of Vi antibodies have been reported, but in the 10-year follow-up^4^ of schoolchildren who were enrolled in a Vi polysaccharide trial in a highly endemic area of South Africa at that time, 58% of children in the control group had acquired measurable levels of Vi antibody over the 10-year period, in keeping with the considerable exposure to the pathogen seen over 2 years in the current study in India. A further line of evidence that these changes in Vi antibody represent true infection is that the incremental 63% efficacy of the Vi conjugate compared to Vi polysaccharide identified in this study translates to 85% protection against typhoid fever using an estimate of Vi polysaccharide baseline protection of 59% [5]. This estimate of efficacy is consistent with the only efficacy study to date comparing a Vi conjugate to placebo in Vietnam (91% efficacy against blood culture proven typhoid fever [6]), which strengthens the argument that this efficacy estimate effect is protection against true S. Typhi exposures.

There are more things to consider about typhoid fever that arise from this study than just Vi conjugate vaccine efficacy and the frequency of subclinical typhoid exposure in endemic areas.

The asymptotic line in Figure 21 suggests that even considerable levels of Vi antibody will still not completely protect against subsequent typhoid infection. These subjects may or may not be clinically ill, but the degree to which one or more typhoid exposures defined by alterations in their Vi antibodies over time, leads to protection from infection in future time periods, could be calculated in future descriptive sero-epidemiological time series in typhoid endemic areas to assess in endemic areas, the protection afforded by the disease itself.

A key issue raised in this study is a correlate of protection. Although no step function was found, there are few data to suggest such a step function of protection from any conjugate vaccines, and the correlate chosen is unlikely to be an absolute correlate. It is more useful to ask the question—if this study suggests 85% protection from typhoid infection, what level of antibody at 42 days postimmunization was achieved by 85% of vaccinees? This is not directly addressed in the article, but Figure 2 suggests its somewhere above 1000 Elisa units. Indeed, very few of the cases presumed to have been infected with S. Typhi before 18 months postimmunization had 42 day levels above 1000 EU (revised Figures 1B and D). (The figures are wrongly labeled in the Voysey paper – according to the legend and the data themselves 1B legend is actually for Figure 1D, so that 1D Figure should be 1B; the current 1B figure should be 1C and the current 1C figure should be 1D.) Future conjugate Vi vaccines could be licensed based on their ability to elicit antibodies equivalent to those found among 85% of vaccinees in this study provided that the assay is benchmarked against a standard serum [7]. An analysis of the Vietnam trial long-term Vi antibody persistence suggested that Vi antibodies postimmunization need to be sufficient to achieve long-term concentrations above 1.4 µg/mL^8^, similar to the estimate of 1 ug/mL required for protection post Vi polysaccharide in the South African trial [9].

Finally, although these data suggest that the greater immunogenicity of Vi conjugates are associated both with increased rates of protection and also with longer duration of protection from S. Typhi infections, than are polysaccharide Vi vaccines, the question of protection of young infants could not be directly addressed from this small study as there was no control group and the attack rate in young infants may be lower than that in the older age groups. Similar small immunogenicity studies with control groups and two or more years of follow-up in endemic areas should quickly be able to address this question.

This study supports the recent World Health Organization Strategic Advisory Group of Experts decision to recommend the use of Vi conjugate vaccine as an exciting new tool to control typhoid fever in endemic areas and an assessment of the Vi antibody status of young people in those areas could be a key opportunity to define the current areas of need.

## Note

***Potential conflicts of interest***. The author: No reported conflicts of interest. The author has submitted the ICMJE Form for Disclosure of Potential Conflicts of Interest. Conflicts that the editors consider relevant to the content of the manuscript have been disclosed.
